# Supplementary material for: Bright split red fluorescent proteins for the visualization of endogenous proteins and synapses
Source: Commun Biol. 2019 Sep 17;2:344. doi: 10.1038/s42003-019-0589-x (PMC6749000; doi:10.1038/s42003-019-0589-x)
Supplement: Supplementary file 1 — Supplementary Material [file 42003_2019_589_MOESM1_ESM.pdf]

Supplementary Figures

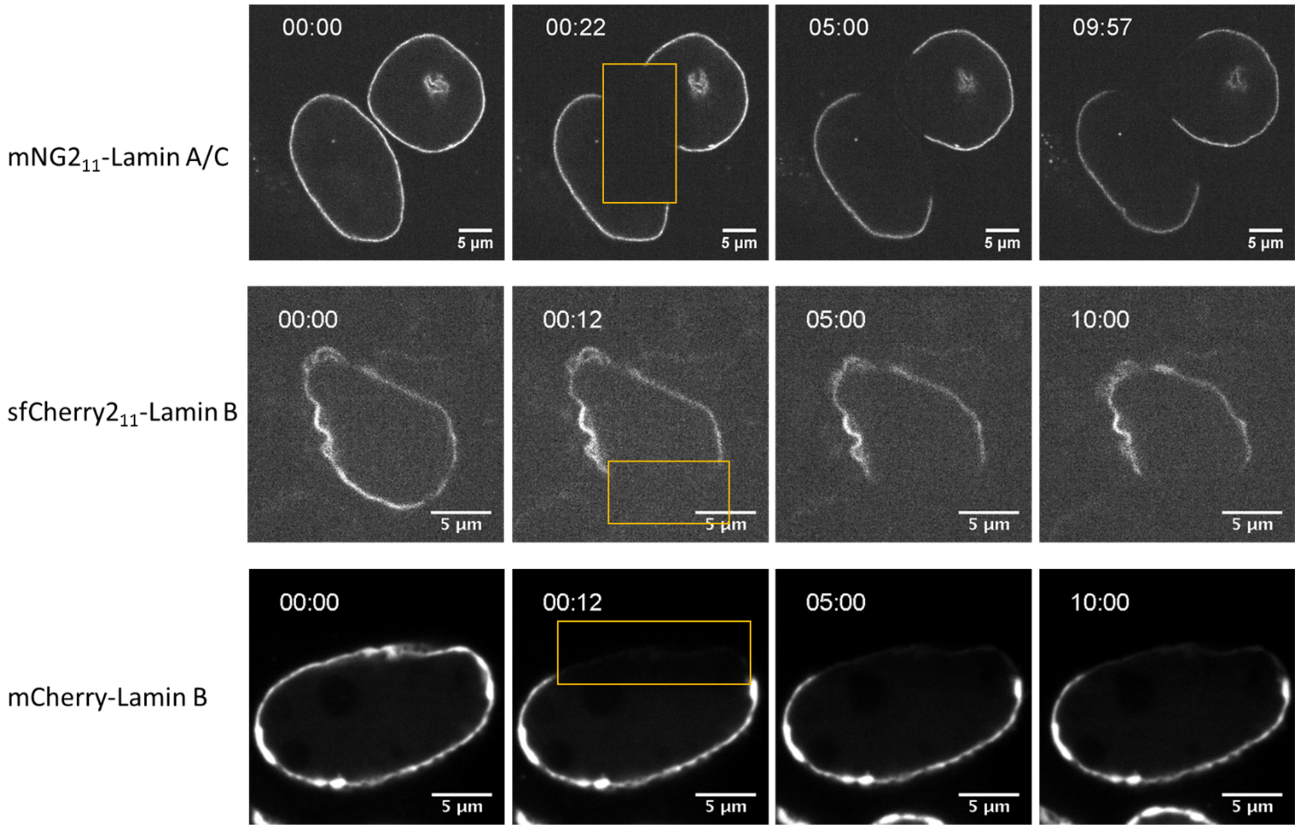

Supplementary Figure 1. Snapshots of fluorescence recovery after photobleaching (FRAP) experiment of endogenously mNeonGreen2$_{1\text{-}10/11}$ labeled Lamin A/C, over-expressed sfCherry2$_{1\text{-}10/11}$ labeled Lamin B, or over-expressed mCherry labeled Lamin B as a positive control in HEK 293T cells at different time points (before bleaching, 1st frame, 5 min and 10 min after bleaching). The yellow rectangular marks the photobleaching area.

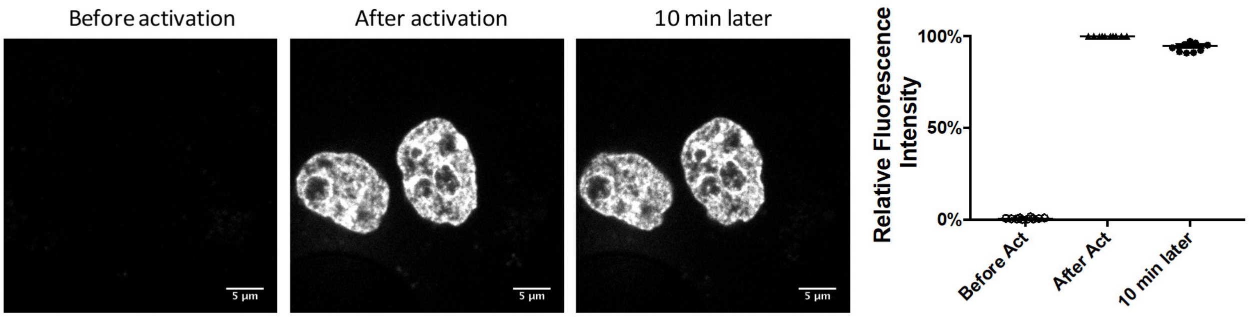

Supplementary Figure 2. Confocal microscopy images of photoactivation in HEK 293T cells co-transfected with sfCherry2$_{11}$-H2B and PAsfCherry2$_{1\text{–}10}$. Three time points (before photoactivation, right after photoactivation and 10 min after photoactivation) were recorded. Mean fluorescence intensities within the nucleus were quantified and normalized to the intensity of the same cell right after photoactivation. $N = 10$ cells in each case.

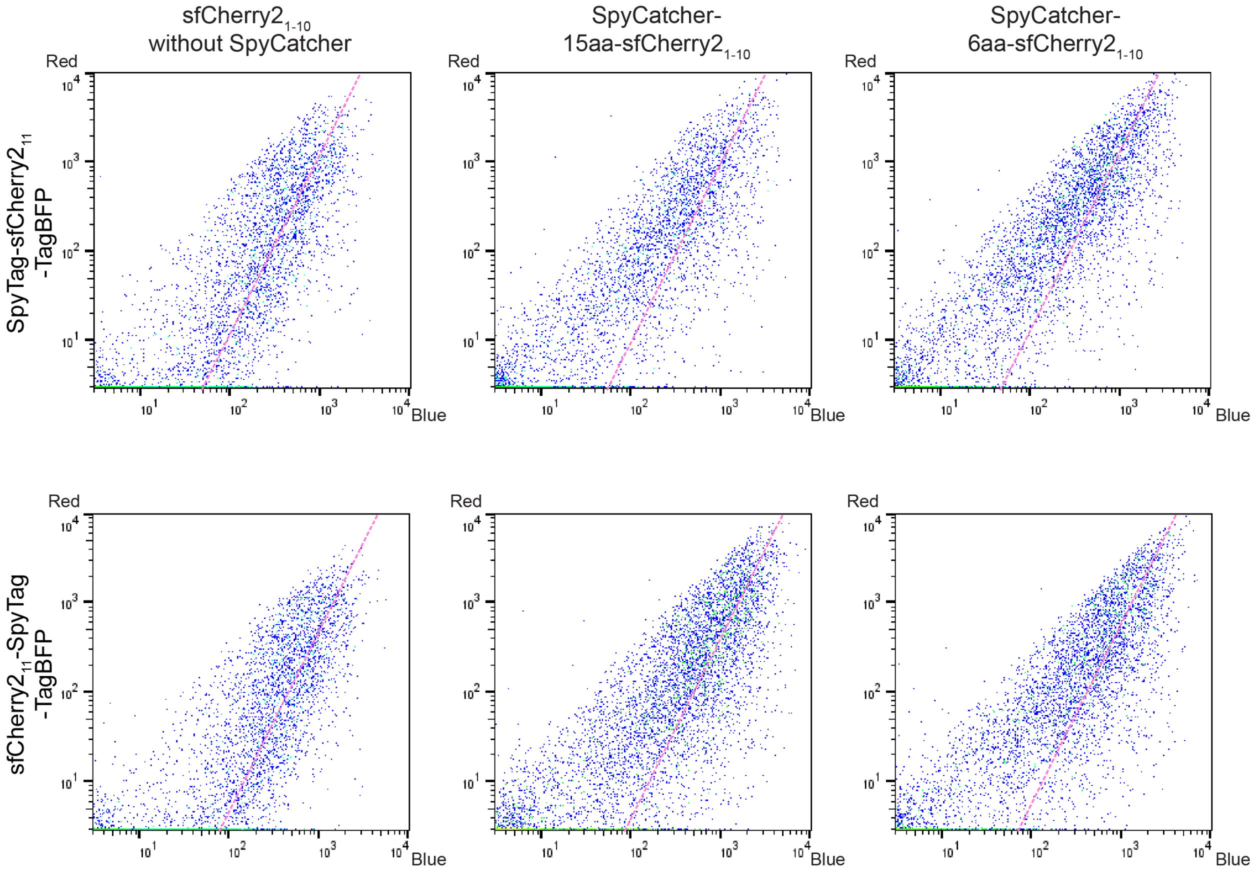

Supplementary Figure 3. Flow cytometry analysis of whole cell fluorescence in HEK 293T cells expressing different tandem tags (SpyTag-sfCherry2$_{11}$-TagBFP and sfCherry2$_{11}$- SpyTag-TagBFP) with different binders (sfCherry2$_{1-10}$ alone, SpyCatcher-15aa-sfCherry2$_{1-10}$ and SpyCatcher-6aa-sfCherry2$_{1-10}$). The pink dashed trend line has a slope of 2.

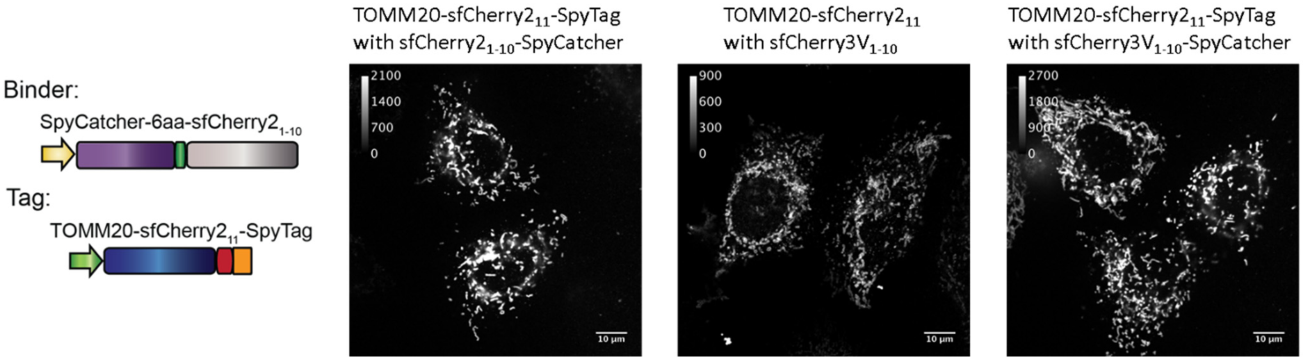

Supplementary Figure 4. Schematics and confocal microscopy images of C-terminal labeled TOMM20 with or without the assistance of SpyTag/SpyCatcher interaction in live HeLa cells using sfCherry2$_{1-10}$ with SpyCatcher, sfCherry3V$_{1-10}$ without SpyCatcher or sfCherry3V$_{1-10}$ with SpyCatcher. Calibration bar shows the fluorescence intensity in each image.

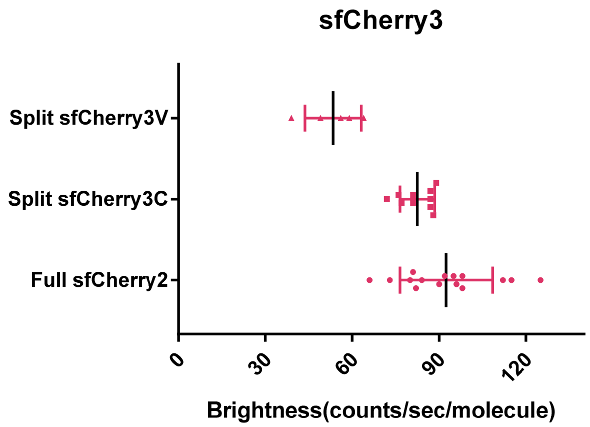

Supplementary Figure 5. Single-molecule brightness measurement of split sfCherry3V, split sfCherry3Cs and full-length sfCherry2 using fluorescence fluctuation spectroscopy by two-photon excitation. $N = 5$, 10 and 15 measurements for sfCherry3V, sfCherry3C and sfCherry2, respectively.

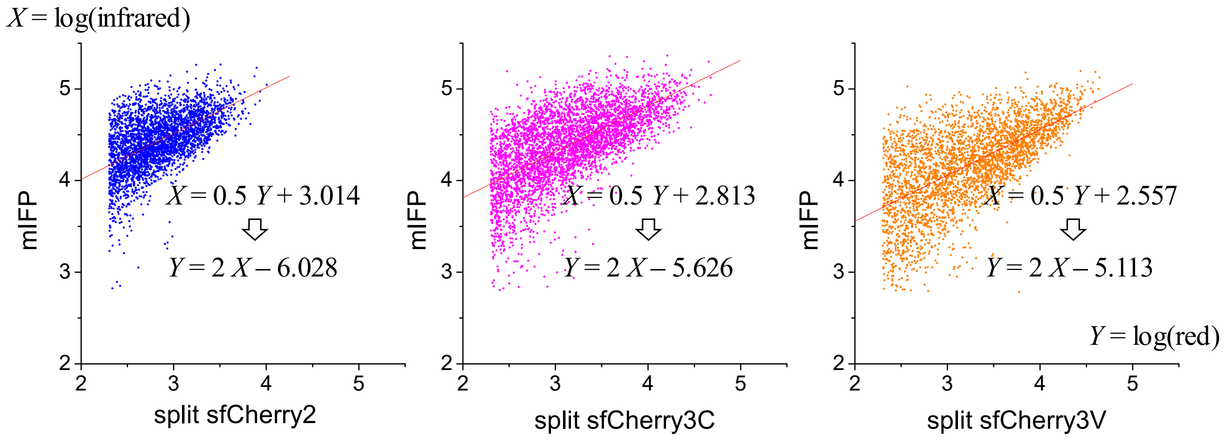

Supplementary Figure 6. Least square linear fitting of flow cytometry points in Figure 3 E with a fixed slope of 0.5. The *x*- and *y*-axes were flipped compared to main figures in order to properly handle the asymmetric errors in the two channels. Points with intensities within 3σ of the scattering background in either channel were excluded from the fitting. The intercept change in the fitted line (from 3.014 ± 0.004 to 2.813 ± 0.005 to 2.557 ± 0.006, ± standard error for least square fitting) was used to calculate the relative change in dissociation constant K_D' (see Supplementary Notes).

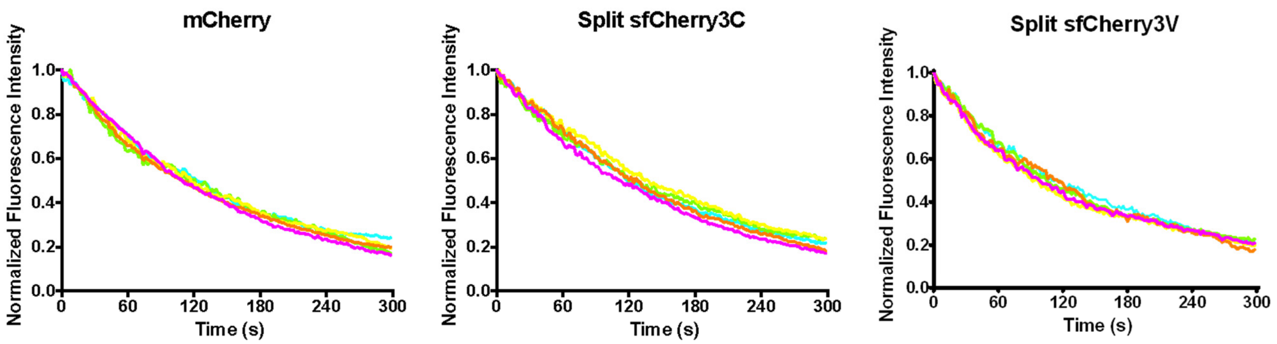

Fluorescent Proteins	Full length mCherry	Split sfCherry3C	Split sfCherry3V
Photobleaching Half-life (s)	98.8 ± 2.9 (S.E.M.)	120.6 ± 6.8 (S.E.M.)	85.1 ± 5.6 (S.E.M.)

Supplementary Figure 7. Fluorescence photobleaching time traces and analysis of photobleaching half-life of split sfCherry3C/3V and full length mCherry in living mammalian cells, obtained by fitting with a single-exponential decay function. Five cells were analyzed in each condition, each trace represents one cell.

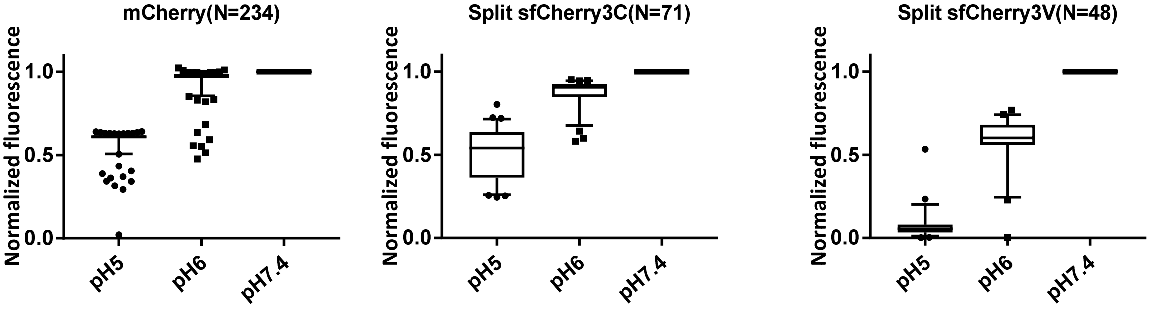

Supplementary Figure 8. pH stability of split sfCherry3 variants compared with full length mCherry in fixed HEK 293T cells at pH = 5, 6, or 7.4 respectively. The fluorescence of individual cells was normalized to the value at pH = 7.4 for the same cell. The error bars are standard error of means. The pKa was measured for each fluorescent protein by fitting points with a titration curve, yielding 4.8 for mCherry, 5.0 for split sfCherry3C and 5.9 for split sfCherry3V. N stands for the number of cells measured. Box-plots: center line, median; box limits, 25th and 75th quartiles; whiskers, 1.5× interquartile range; points, outliers.

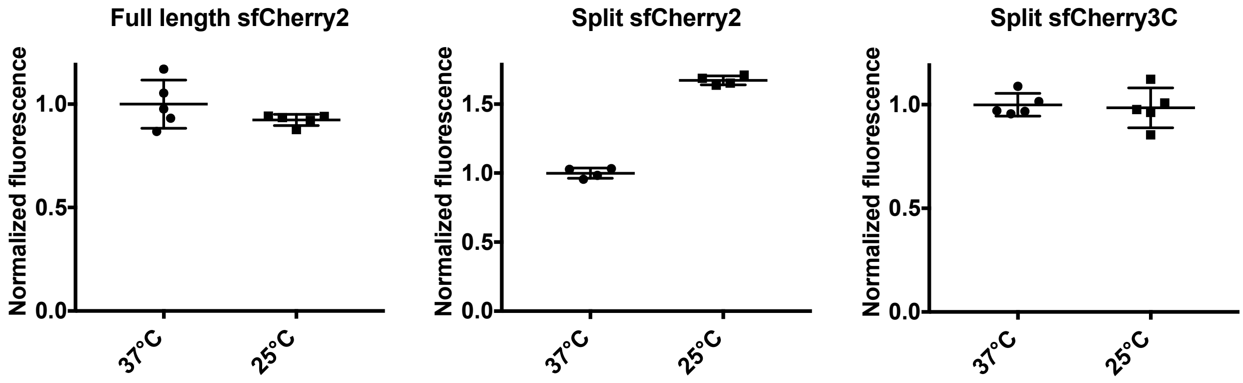

Supplementary Figure 9. Temperature stability of full length sfCherry2, split sfCherry2 and split sfCherry3C expressing in live *E. coli* cells at 37 °C and 25 °C. The fluorescence intensity for each *E. coli* culture was first normalized to the cell density (OD600) and then normalized to its intensity at 37 °C. The error bars are standard deviations. For full length sfCherry2 and split sfCherry3C, $N = 5$ *E. coli* cultures; for split sfCherry2, $N = 4$ *E. coli* cultures.

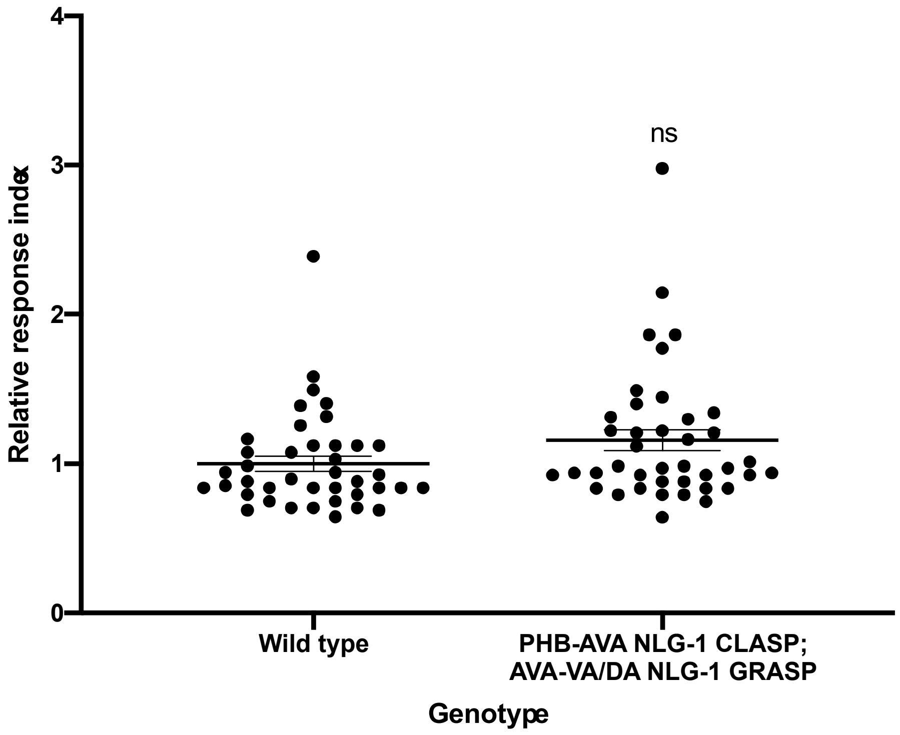

Supplementary Figure 10. PHB-AVA NLG-1 CLASP does not affect PHB circuit function. Dot plot displaying the relative response index to sodium dodecyl sulfate (SDS), a chemical sensed by the PHB circuit. An increase in synaptogenesis between PHB and AVA neurons should cause a decrease in the response time, and a corresponding decrease in the relative response index. However, wild type animals carrying the PHB-AVA NLG-1 CLASP and AVA-VA/DA NLG-1 GRASP markers have a response index that is not significantly different from that of wild type animals not carrying the markers. See Materials and Methods for a more detailed description of the assay. N = 40 animals for each group, ns P > 0.05, t-test, comparison with wild type animals not carrying the markers.

Supplementary Tables

Supplementary Table 1: Sequences of sfCherry3C$_{1\text{-}10}$, sfCherry3V$_{1\text{-}10}$, SpyCatcher 002 and SpyTag 002.

	DNA Sequence
**sfCherry3C$_{1\text{-}10}$**	GAGGAGGACAACATGGCCATCATCAAGGAGTTCATGAGATTCAAGGTG<br>CACATGGAGGGCAGCGTGAACGGCCACGAGTTCGAGATCGAGGGCGA<br>GGGCGAGGGCCACCCCTACGAGGGCACCCAGACCGCCAGGCTGAAGGT<br>GACCAAGGGCGACCCCCTGCCCTTCGCCTGGGACATCCTGAGCCCCCA<br>GTTCATGTACGGCAGCAAGGCCTACGTGAAGCACCCCGCCGACATCCC<br>CGACTACCTGAAGCTGAGCTTCCCCGAGGGCTTCACCTGGGAGAGAGT<br>GATGAACTTCGAGGACGGCGGCGTGGTGGCCGTGACCCAGGACAGCAG<br>CCTGCAGGACGGCCAGTTCATCTACAAGGTGAAGCTGCTGGGCATCAA<br>CTTCCCCAGCGACGGCCCCGTGATGCAGAAGAAGACCATGGGCTGGGA<br>GGCCAGCACCGAGAGAATGTACCCCGAGGACGGCGCCCTGAAGGGCG<br>AGATCAACCAGAGACTGAAGCTGAAGGACGGCGGTCACTACGACGCCG<br>AGGTGAAGACCACCTACAGGGCCAAGAAGCCCGTGCAGCTGCCCGGCG<br>CCTACGACGTGGACATCAAGCTGGACATCACCAGCCACAACGAGGAC
**sfCherry3V$_{1\text{-}10}$**	GAGGAGGACAACATGGCCATCATCAAGGAGTTCATGAGATTCAAGGTG<br>CACATGGAGGGCAGCGTGAACGGCCACGAGTTCGAGATCGAGGGCGA<br>GGGCGAGGGCCACCCCTACGAGGGCACCCAGACCGCCAGGCTGAAGGT<br>GACCAAGGGCGACCCCCTGCCCTTCGCCTGGGACATCCTGAGCCCCCA<br>GTTCATGTACGGCAGCAAGGCCTACGTGAAGCACCCCGCCGACATCCC<br>CGACTACTGGAAGCTGAGCTTCCCCGAGGGCTTCACCTGGGAGAGAGT<br>GATGAACTTCGAGGACGGCGGCGTGGTGGCCGTGACCCAGGACAGCAG<br>CCTGCAGGACGGCCAGTTCATCTACAAGGTGAAGCTGCTGGGCATCAA<br>CTTCCCCAGCGACGGCCCCGTGATGCAGAAGAAGACCATGGGCTGGGA<br>GGCCAGCACCGAGAGAATGTACCCCGAGGACGGCGCCCTGAAGGGCG<br>AGATCAACCAGAGACTGAAGCTGAAGGACGGCGGTCACTACGACGCCG<br>AGGTGAAGACCACCTACAGGGCCAAGAAGCCCGTGCAGCTGCCCGGCG<br>CCTACGACGTGGACATCAAGCTGGACATCACCAGCCACAACGAGGAC
**SpyCatcher 002**	GGCGCCATGGTAACCACCTTATCAGGTTTATCAGGTGAGCAAGGTCCGT<br>CCGGTGATATGACAACTGAAGAAGATAGTGCTACCCATATTAAATTCTC<br>AAAACGTGATGAGGACGGCCGTGAGTTAGCTGGTGCAACTATGGAGTT<br>GCGTGATTCATCTGGTAAAACTATTAGTACATGGATTTCAGATGGACAT<br>GTGAAGGATTTCTACCTGTATCCAGGAAAATATACATTTGTCGAAACCG<br>CAGCACCAGACGGTTATGAGGTAGCAACTGCTATTACCTTTACAGTTAA<br>TGAGCAAGGTCAGGTTACTGTAAATGGCGAAGCAACTAAAGGTGACGC<br>TCATACT
**SpyTag 002**	GTGCCTACTATCGTGATGGTGGACGCCTACAAGCGTTACAAG

Supplementary Table 2: DNA sequences used for sgRNA in vitro transcription

Target Gene	Target Term	Sequence of DNA oligo for sgRNA synthesis
LMNA	N	TAATACGACTCACTATAGGCCATGGAGACCCCGTCCCAGGTTTAAGAGCTATGCTGGAA
CLTA	N	TAATACGACTCACTATAGGGCCATGGCGGGCAACTGAAGTTTAAGAGCTATGCTGGAA
RAB11A	N	TAATACGACTCACTATAGGGTAGTCGTACTCGTCGTCGGTTTAAGAGCTATGCTGGAA
HP1b	N	TAATACGACTCACTATAGGAAAGCTGGCGGGCACTATGGTTTAAGAGCTATGCTGGAA
SEC61b	N	TAATACGACTCACTATAGGCTTGTCTCCCTCTACAGCCGTTTAAGAGCTATGCTGGAA
ARL6IP1	N	TAATACGACTCACTATAGGATCCCCGAGACGATGGCGGGTTTAAGAGCTATGCTGGAA

Supplementary Table 3: Oligo-nucleotide donor DNA sequences

Target Gene-FP_{11} Tag	DNA sequence
LMNA-sfCherry2_{11}	TCCTTCGACCCGAGCCCCGCGCCCTTTCCGGGACCCCTGCCCCGC<br>GGGCAGCGCTGCCAACCTGCCGGCCATGTACACCATCGTGGAGC<br>AGTACGAGAGAGCCGAGGCCAGACACAGCACCGGTGGCGGCGA<br>GACCCCGTCCCAGCGGCGCGCCACCCGCAGCGGGGCGCAGGCCA<br>GCTCCACTCCGCTGTCGCCCACCC
CLTA-sfCherry2_{11}	CGGGCGTGGTGTCGGTGGGTCGGTTGGTTTTTGTCTCACCGTTGG<br>TGTCCGTGCCGTTCAGTTGCCCGCCATGTACACCATCGTGGAGCA<br>GTACGAGAGAGCCGAGGCCAGACACAGCACCGGAGGTGGCATG<br>GCTGAGCTGGATCCGTTCGGCGCCCCTGCCGGCGCCCCTGGCGGT<br>CCCGCGCTGGGGAACGGAGTGG
RAB11A-sfCherry2_{11}	CCCTGCAGCGACGCCCCCTGGTCCCACAGATACCACTGCTGCTCC<br>CGCCCTTTCGCTCCTCGGCCGCGCAATGTACACCATCGTGGAGCA<br>GTACGAGAGAGCCGAGGCCAGACACAGCACCGGAGGTGGCGGC<br>ACCCGCGACGACGAGTACGACTACCTCTTTAAAGGTGAGGCCAT<br>GGGCTCTCGCACTCTACACAGTC
HP1b-sfCherry2_{11}	CTAATGCCCTTTTTATTTCATTTATCATTTTAGCAGCGTCACCCTT<br>TACACCAGAAAGCTGGCGGGCACTATGTACACCATCGTGGAGCA<br>GTACGAGAGAGCCGAGGCCAGACACAGCACCGGTGGCGGCGGG<br>AAAAAACAAAACAAGAAGAAAGTGGAGGAGGTGCTAGAAGAGG<br>AGGAAGAGGAATATGTGGTGGAAA
SEC61b-sfCherry2_{11}	GTGTCTAGGCCGGGGTTCTGGGGCAGGCCTGCCGCGCTCACCCG<br>TCTGTCTGCTTGTCTCCCTCTACAGTACACCATCGTGGAGCAGTA<br>CGAGAGAGCCGAGGCCAGACACAGCACCGGTGGCGGCCCTGGTC<br>CGACCCCCAGTGGCACTAACGTGGGATCCTCAGGGCGCTCTCCC<br>AGCAAAGCAGTGGCCGCCCGGGC
ARL6IP1-sfCherry2_{11}	GCGGGTTTCGGTTGGAGGACTCGTTGGGGAGGTGGCCTGCGCTT<br>GTAGAGACTGCATCCCCGAGACGATGTACACCATCGTGGAGCAG<br>TACGAGAGAGCCGAGGCCAGACACAGCACCGGTGGCGGCGCGG<br>AGGGAGATAATCGCAGCACCAACCTGCTGGTGAGTCCTGGCTGC<br>CTGTCCCCCGGGAGCCGAGCGA

sfCherry2_{11} sequence
Linker sequence
Coding region sequence

Supplementary Note

A two-step model for $FP_{1-10/11}$ complementation

The overall complementation process of commonly used split fluorescent proteins is known to be irreversible [1, 2, 3], raising the possibility that the complementation of split $mNG2_{1-10/11}$ and split $sfCherry2_{1-10/11}$ is also irreversible. Here, we verified this possibility by fluorescence recovery after photobleaching (FRAP) experiment. In HEK 293T cells with lamin A/C endogenously labeled by $mNG2_{11}$ and constitutively overexpressed $mNG2_{1-10}$ [4], bleaching a part of the nuclear lamina led to no observed fluorescence recovery in 10 min (Supplementary Fig. 1). Considering the fast maturation of mNeonGreen (< 10 min) [5], this lack of fluorescence recovery indicated that there was little exchange between the complemented $mNG2_{1-10}$ with free cytosolic $mNG2_{1-10}$. We also validated the irreversible complementation for $sfCherry2_{1-10/11}$ by similar FRAP experiment in HEK 293T cells overexpressing $sfCherry2_{11}$ labeled lamin A and $sfCherry2_{1-10}$ (Supplementary Fig. 1). FRAP experiment of mCherry labeled lamin A was used as a positive control (Supplementary Fig. 1). To rule out the possibility of such a lack of recovery was caused by photobleaching-induced damaging of the FP_{11} fragment, we performed the inverse experiment of photoactivation using a photoactivatable variant of $sfCherry2_{1-10}$ [4] (Supplementary Fig. 2). In this case, < 5% fluorescence reduction was recorded at 10 min after photoactivation, confirming that fragment exchange after complementation, even if indeed happening, is at a very slow rate.

On the other hand, a directly irreversible complementation would result in a proportional relationship between the complemented fluorescence signal and the fragment expression level. In this case, the flow cytometry data points in Figure 1C should follow a trend line with a slope of 1, whereas in reality, they fall closer to a trend line with a slope of 2. To explain this observation, we consider a two-step complementation model, in which the first step is a relatively fast reversible binding between the FP_{1-10} and FP_{11} fragments, forming an intermediate $FP^*_{1-10/11}$ complex, followed by a second, slower step of irreversible folding and/or fluorophore maturation.

$$\mathrm{FP}_{1-10} + \mathrm{FP}_{11} \underset{\text{binding}}{\overset{K_D}{\rightleftharpoons}} \mathrm{FP}^*_{1-10/11} \xrightarrow[\text{folding/maturation}]{k_f} \mathrm{FP}_{1-10/11} \xrightarrow{k_{deg}} \text{degradation}$$

At a steady state, the concentration of the complemented, functionally fluorescent species, $FP_{1-10/11}$, is related to the concentration of the intermediate complex, $FP^*_{1-10/11}$, by:

$$[\mathrm{FP}_{1-10/11}] = \frac{k_f}{k_{deg}}[\mathrm{FP}^*_{1-10/11}]$$

where k_f and k_{deg} are the folding/maturation rate and degradation rate, respectively. Generally, we would expect that the folding rate is much faster than the degradation rate, i.e. $k_f >> k_{deg}$ and hence $[\mathrm{FP}_{1-10/11}] >> [\mathrm{FP}^*_{1-10/11}]$. The concentration of $FP^*_{1-10/11}$ is further connected to the concentration of the free fragments as:

$$[\mathrm{FP}^*_{1-10/11}] = \frac{1}{K_D}[\mathrm{FP}_{1-10}][\mathrm{FP}_{11}]$$

where K_D is the dissociation constant for the initial binding step. In our experiment, FP_{11} and $FP_{1\text{-}10}$ are expressed from co-transfected plasmids. Figure 1E indicates that their expression levels in individual cells follow a proportional relationship:

$$[\mathrm{FP}_{1-10}] = a[\mathrm{FP}_{11}]$$

where a should be close to 1 considering that the same expression vectors are used for FP_{11} and $FP_{1\text{-}10}$. Taken together, we have:

$$\left[\mathrm{FP}_{1-10/11}\right] = \frac{1}{K_D{}'}[\mathrm{FP}_{11}]^2$$

where the effective dissociation constant

$$K_D{}' = \frac{k_{deg}}{k_f a} K_D$$

In our flow cytometry experiment, the complemented fluorescence signal, Y, is proportional to $\left[\mathrm{FP}_{1-10/11}\right]$, whereas the expression marker (mIFP) signal, X, is actually proportional to the total concentration of complemented and uncomplemented FP_{11} fragment

$$X \propto \left[\mathrm{FP}_{1-10/11}\right] + \left[\mathrm{FP}^{*}_{1-10/11}\right] + [\mathrm{FP}_{11}] \approx \left[\mathrm{FP}_{1-10/11}\right] + [\mathrm{FP}_{11}] = \left[\mathrm{FP}_{1-10/11}\right] + \sqrt{K_D{}'\left[\mathrm{FP}_{1-10/11}\right]}$$

considering that $\left[\mathrm{FP}_{1-10/11}\right] >> \left[\mathrm{FP}^{*}_{1-10/11}\right]$.

The relationship between Y and X, on a log-log plot, follows the black curve on the simulated plot below:

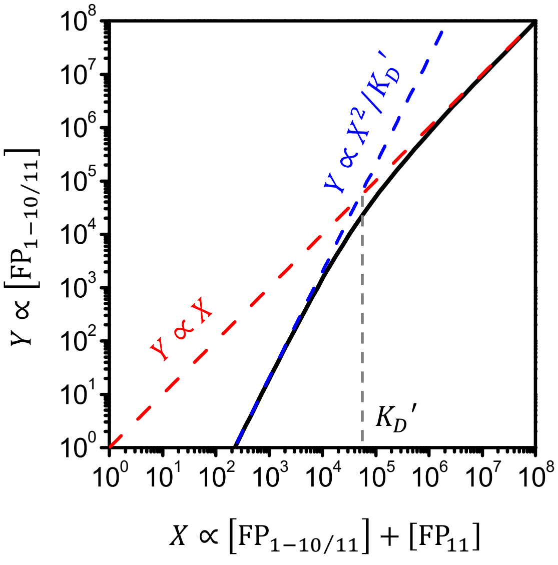

At a high fragment expression level or with a small $K_D{}'$ (the case of $GFP_{1\text{-}10/11}$), X is dominated by the first term of $\left[\mathrm{FP}_{1-10/11}\right]$, making the right end of the curve approaching a slope of 1. On the other hand, at a low fragment expression level or with a large $K_D{}'$ (the cases of $mNG2_{1\text{-}10/11}$ and $sfCherry2_{1\text{-}10/11}$), X is

dominated by the second term of $[FP_{11}] = \sqrt{K_D'[FP_{1-10/11}]}$, making the left end of the curve approaching a slope of 2.

In this latter regime, only a small fraction of the FP_{11} fragment is complemented with the FP_{1-10} fragment to reconstitute a functional fluorescent protein. Consequently, the apparent brightness of the FP_{11}-labeled protein is lower than protein labeled by the full length FP. The complementation efficiency, defined as the fraction of FP_{11} complemented with FP_{1-10}, is directly proportional to the concentration of FP_{1-10}:

$$\text{complementation efficiency} = \frac{[FP_{1-10/11}]}{[FP_{1-10/11}] + [FP^*_{1-10/11}] + [FP_{11}]} \approx \frac{[FP_{1-10/11}]}{[FP_{11}]} = \frac{[FP_{1-10}]}{K_D''}$$

where $K_D'' = aK_D'$ (i.e. effective dissociation constant with respect to FP_{1-10} instead of FP_{11}). This relationship holds true both for our FACS experiment and for any general applications of FP_{11} labeling. In other words, the complementation efficiency improves with higher expression level of the FP_{1-10} fragment. Therefore, overexpression of $mNG2_{1-10}$ and $sfCherry2_{1-10}$ improves the apparent brightness of the FP_{11} labeled proteins. This improvement saturates when the concentration of FP_{1-10} is approaching K_D''. Therefore, a trade-off must be considered between the diminishing brightness improvement and the potential cytotoxicity of high FP_{1-10} expression.

Obviously, a better approach to improve the apparent brightness / complementation efficiency is to reduce K_D' (equivalent to reducing K_D''). This is exactly what we tried to achieve by engineering sfCherry3C and sfCherry3V. In these cases, the improvement of K_D' can be measured by fitting the FACS data with a line at a slope of 2 and then measure the change in the intercept (Fig. S6).

From sfCherry2 to sfCherry3C, the K_D' decreased by:

$$\frac{K_D'(\text{sfCherry2})}{K_D'(\text{sfCherry3C})} = 10^{6.028-5.628} = 2.5$$

From sfCherry2 to sfCherry3V, the K_D' decreased by:

$$\frac{K_D'(\text{sfCherry2})}{K_D'(\text{sfCherry3V})} = 10^{6.028-5.113} = 8.2$$

Based on the schematic figure above, using flow cytometry scatter plot, we can further estimate the difference in the effective dissociation constant among the various split fluorescent protein constructs we have tested. We were able to perform only a very crude estimation, though, because the expression level range from our transient expression system is limited. For $GFP_{1-10/11}$, the flow cytometry point distribution actually has a slight downward bend away from the slope-1 trend line at the low expression end (Fig. 1B). The position of this bend gives an approximate estimation of the K_D' to be ~ 10^3 (in the units of mIFP fluorescence. Same unit below). For $mNG2_{1-10/11}$, the full-length (slope = 1) and split (slope = 2) trend lines cross at approximately $K_D' = 2 \times 10^5$ (Fig. 1C). For $sfCherry2_{1-10/11}$, the full-length (slope = 1) and split (slope = 2) trend lines cross at approximately $K_D' = 3 \times 10^5$ (Fig. 1D). For $sfCherry3V_{1-10/11}$, there is a slight downward bend at the high concentration end (Fig. 3E), giving an approximate K_D' of ~ 3×10^4. Taken together, the effective dissociation constant for $sfCherry3V_{1-10/11}$ is approximately 30 times of that of $GFP_{1-10/11}$, compared to ~ 300 times in the case of $sfCherry2_{1-10/11}$. This ~ 10 fold improvement is consistent with our direct comparison (8.2 fold) measured in Figure 3E.

References

1. Cabantous, S. and G.S. Waldo, In vivo and in vitro protein solubility assays using split GFP. *Nat Methods* **3**, 845-854 (2006).
2. Kerppola, T.K., Visualization of molecular interactions by fluorescence complementation. *Nat Rev Mol Cell Biol* **7**, 449-456 (2006).
3. Köker, T., A. Fernandez, and F. Pinaud, Characterization of Split Fluorescent Protein Variants and Quantitative Analyses of Their Self-Assembly Process. *Scientific Reports* **8**, 5344 (2018).
4. Feng, S., et al., Improved split fluorescent proteins for endogenous protein labeling. *Nat Commun*, **8**, 370 (2017).
5. Shaner, N.C., et al., A bright monomeric green fluorescent protein derived from Branchiostoma lanceolatum. *Nat Methods* **10**, 407-409 (2013).
