# Supplementary material for: Bright split red fluorescent proteins for the visualization of endogenous proteins and synapses
Source: Commun Biol. 2019 Sep 17;2:344. doi: 10.1038/s42003-019-0589-x (PMC6749000; doi:10.1038/s42003-019-0589-x)
Supplement: Supplementary file 2 — Description of Additional Supplementary Files [file 42003_2019_589_MOESM2_ESM.docx]

**Description of Additional Supplementary Files**

**File Name**: **Supplementary Data 1**

**Description**:   The original data for the generation of graphs in Figure 1A, Supplementary Figure 5, Figure 5 and Figure 6
